# Evaluation of a Smartphone Application on the Reduction of Attentional Bias Toward Alcohol Among Students^†^

**DOI:** 10.3389/fpsyg.2022.790030

**Published:** 2022-02-10

**Authors:** Valentin Flaudias, Oulmann Zerhouni, Nadia Chakroun-Baggioni, Ingrid De Chazeron, Pierre-Michel Llorca, Georges Brousse

**Affiliations:** ^1^Université de Nantes, Univ Angers, Laboratoire de Psychologie des Pays de la Loire, LPPL, EA 4638, Nantes, France; ^2^Laboratoire Parisien de Psychologie Sociale, Département de Psychologie, Nanterre, France; ^3^Université Clermont Auvergne, Laboratoire de Psychologie Sociale et Cognitive, Clermont-Ferrand, France; ^4^CMP-B CHU, Clermont Auvergne INP, Institut Pascal, Centre National de la Recherche Scientifique (CNRS), Université Clermont Auvergne, Clermont-Ferrand, France

**Keywords:** attentional bias, alcohol, students, smartphone, prevention

## Abstract

**Context:**

The recent development of “serious games” has produced encouraging results in maintaining adherence to health-related interventions. In alcohol research, several studies have shown that computerized training on attentional bias decreases alcohol consumption bias among students. However, these highly controlled experimental situations, do not allow for direct large-scale dissemination. Our objective is to evaluate an attentional bias remediation program using a gamified smartphone training procedure.

**Methods:**

Fifty students from Clermont-Ferrand University were invited to participate in the study. After a cognitive assessment in the laboratory, the smartphone application was installed on each Student’s smartphone. Participants were randomly assigned to either the alcohol attentional training group or the control group Each student had to complete the 2-min program at least once a day for 15 days. After 15 days, a new cognitive assessment of attention bias was conducted in the laboratory. Forty-seven students were included in the study.

**Results:**

Our analyses did not show any effect of the cognitive remediation program on attentional bias reduction between the two group [*F*_(1, 44)_ < 1, *p* = 0.87], attentional performance [*F*_(1, 45)_ = 1.63, *p* = 0.20] or inhibitory abilities [*F*_(1, 45)_ < 1, *p* = 0.73]. These results were confirmed by Bayesian analyses.

**Discussion:**

Despite the absence of group effects, both the alcohol and control (non-alcohol) version of this program appeared to reduce attentional bias and increase inhibition capacities in the subset of participants who had attentional bias for alcohol at baseline This pilot study identifies areas for improvement in smartphone applications for future developments. Attentional bias remediation programs remain an interesting way to explore.

## Introduction

According to the latest OFDT report (French Observatory of Drugs and Drug Addiction), 40% of 18–75 years old in France drink alcohol regularly with more than 8.4% of 17-year-olds in 2017 will drink alcohol regularly (at least 10 glasses in a month), and 44% will demonstrate at less one binge drinking behavior (4–5 drinks within a specific time period) during the past month (OFDT, 2019). This heavy consumption over less than 2 h can produce destructive short-term effects such as accidents, violence and even alcohol-related comas (Boles and Miotto, 2003) while also impacting spatial working memory (Squeglia et al., 2011) and other cognitive functions (Howland et al., 2010). These negative outcomes underscore the importance of developing tools to prevent problematic alcohol behaviors in young people. University students are at high risk for binge drinking and show higher drinking levels in France as well as in other countries such as the United States of America compared to the general population (White and Hingson, 2014). Students seem particularly vulnerable to alcohol abuse and excessive consumption, which are two risk factors known to increase the risk of future dependence.

Several studies suggest that cognitive factors, such as weaker inhibitory functions (Tarter et al., 2003; Nigg et al., 2004), notably attentional bias (Cox et al., 2002), may be involved in problematic alcohol consumption. Preventive approaches aimed at developing these cognitive functions could therefore be an innovative and effective method to reduce alcohol consumption.

Several tools may be used to study attentional bias. However, the most widely used tool in addiction studies is the alcohol Stroop task. The Alcohol Stroop Test (see Field and Cox (2008) for a review) is an adaptation of the Stroop task that uses alcohol-related words to assess attentional bias: longer response times to name the color of an alcohol-related word than a neutral word indicates an attentional bias toward alcohol (Field and Cox, 2008).

To reduce alcohol attentional bias among young people, Fadardi and Cox (2009) developed a cognitive training program to bolster attentional control in students with problematic alcohol behaviors. In this study, students were trained on an alcohol-related attention task, the Alcohol Attention Control Training program, to assess its impact on attentional bias. Participants were asked to complete series of exercises on a computer. In the first series, bottles of alcoholic and non-alcoholic beverages were individually and randomly displayed on the screen against a colored background (blue, yellow, red, or green). The participant was then asked to name the background color. In a second series, the bottle pictures were surrounded by a colored frame. The participant was asked to name the color of the frame. Finally, in the last series, two pictures were presented: one of a bottle of an alcoholic drink and one of a non-alcoholic drink, each picture being surrounded by a different colored frame. The participant was then asked to name the color of the frame of the non-alcoholic bottle. The results of the study showed that at the start of the program, students who were regular drinkers showed higher attentional bias compared to occasional drinkers. While training reduced the attentional bias of both occasional and regular drinkers, regular drinkers also decreased their alcohol consumption. Moreover, these improvements were maintained 3 months later.

Because this Alcohol Attention Control Training program requires a long time doing a daunting task on a computer, it is difficult to apply this tool to settings outside of research laboratories and is therefore not suitable for wide dissemination. The objective of the study proposed here is to validate a program for the cognitive remediation of attentional bias in students who occasionally or regularly consume alcohol. To do this, we developed a smartphone game that would be easily accessible to students; the game had a short duration (less than 2 min) but repeated several times. This smartphone program was an adapted version of a previously program developed and described elsewhere (Flaudias et al., 2019), to make it more attractive to students.

There are several advantages of smartphones compared to traditional computers. For example, Klasnja and Pratt (2012) noted that anonymity, portability, and ease of access and use are appealing. Moreover, the smartphone uses double encoding which allows better memorization due to movement of the arm and finger (see Denham et al. (2012) for an embodied approach of mobile learning). The smartphone is also more ecologically relevant because it uses real movements. In addition, it is a widespread tool: according to a recent report, 98% of 18–24 year old have a smartphone (Statista, 2019).

This study aims to reduce the attentional bias of students who drink alcohol via a smartphone application. The alcohol-training group was contrasted with a control group who did the same attentional training, but toward fruit and vegetables. In this pilot study, alcohol attentional bias was expected to decrease after 15 days of using the alcohol-related program, but not for the control group that used the fruit and vegetables program. Furthermore, this decrease was expected to be independent of any improvement in attention span.

## Materials and Methods

### Participants and Procedure

Fifty students were invited to the Social and Cognitive Psychology Laboratory of Clermont-Ferrand to evaluate an application on health related issues on smartphone in exchange for a course credit. The students have been informed about this study via the bulletin boards specifically concerning the studies conducted in the laboratory. Participants were randomly assigned to either the alcohol attentional training group or the control group. The participant was required to play the attentional program for 2 min each day for a period of 15 days. Daily notification reminded the participant to play at the end of the day if playing had not yet occurred. During this first interview, the participants were evaluated on their level high-risk alcohol use, their level of craving, their capacities of inhibition and their attentional bias. After the 15 days, participants returned to the laboratory to perform attention tests again to assess attentional bias and inhibition capacities. This study was approved by the Committee for the Protection of Persons. Based on a previous study on attentional bias (Flaudias et al., 2019), considering a mean standard deviation of 38 ms for groups with an initial attentional bias, reduction of attentional bias by half after the program, i.e., 58.5 ms, for a α = 0.05 and a β = 0.8 risk, we would need 9 participants per group.

### Measures

#### Self-Complete Questionnaires Assessing the Level of Problem Alcohol Use

Alcohol Use Disorders Identification Test (AUDIT) (Saunders et al., 1993). This questionnaire is a self-questionnaire developed by the World Health Organization (WHO) and has been validated in both general and specific populations. This tool is used to identify participants with high-risk alcohol use and consists of 10 items, rated from 0 to 4. A score greater than or equal to 8 in men and 7 in women suggests alcohol misuse. A score greater than 12 for males and greater than 11 for females is suggestive of alcohol dependence (Rubinsky et al., 2010). The AUDIT explores three dimensions: frequency and quantity of alcohol consumed, dependence, and problems encountered resulting from alcohol consumption. The original alpha of Cronbach was between 0.93 and 0.81. It was at 0.811 for our study.

Obsessive compulsive drinking scale (OCDS) (Anton et al., 1995). This 14-item Alcoholic Appetence Self-Appetitiveness Questionnaire is a French translation of the OCDS. It is a quick and easy-to-use instrument that offers good validity, reliability, and internal consistency (Chignon et al., 1997). The original alpha of Cronbach was between 0.89. It was at 0.885 for our study.

#### Cognitive Tests of Attentional Bias and Attention Skills

Alcohol Stroop Test (AST). We used the same Alcohol Stroop Test (AST) as Flaudias et al. (2013) to assess attentional bias toward alcohol. In this computerized task, participants were asked to name verbally the color of three categories of words: alcohol (e.g., “beer”), color (e.g., blue), and neutral words (e.g., table). The response times were recorded by a microphone on the PC. Participants were instructed to concentrate on the fixation cross (+) for 500 ms before it was replaced by the word. Stimuli were presented in blocks to avoid the cognitive influence of the item “alcohol” being carried over to the following item. The experiment began with a set of two practice blocks. The three blocks were presented in random order between participants, with a pause of a few seconds between each block. In total, except for practice items, each participant saw 72 items. The words were presented in three different colors, twice for each block. Attentional control was assessed with the classic Stroop test (difference in response time between colored and neutral words). Attentional bias was calculated by subtracting the response times between alcohol words and neutral words. An attention score was also calculated by subtracting the response times of colored and neutral words. The Stroop task has long been known to be unaffected by test-retest effects over periods ranging from 1 to 2 weeks (Franzen et al., 1987).

Hayling test (Burgess and Shallice, 1996). The Hayling test measures the ability to inhibit a semantic response that is automatically actived but not appropriate for the task. It consists of two sets of 15 sentences each having the last word missing. In the first part of this test, the participant must complete a sentence with a semantically appropriate word (Part A). For example “The old house will be torn… (down).” In the second part of this task, which is the inhibition phase, a sentence must be completed with a semantically unrelated word (Phase B). For example “The captain wanted to stay with the sinking…(e.g., light bulb).” Three scores are calculated: an initiation score which is the total time to respond to the 15 sentences of phase A, an inhibition score which corresponds to the total time of phase B, and, finally, an interference score which corresponds to the difference between the score in phase B and phase A. A higher score indicates lower inhibition capabilities. To avoid a test-retest effect in this evaluation, the sentences used for phase A and B were randomized between participants.

### Description of the Cognitive Remediation Program on Smartphone

**FIGURE 1:**
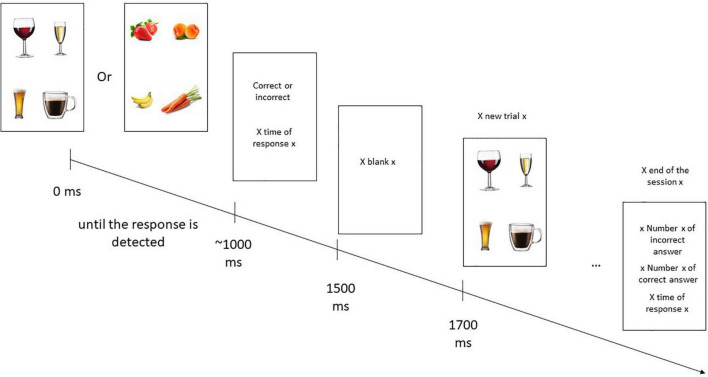
Presentation of the cognitive remediation program on a smartphone.

For each smartphone, the participant was instructed to press the presented item that was not alcohol-related (or was not a fruit for the control condition) as quickly as possible. On the screen, four images were displayed: three alcohol-related (or fruit) and one unrelated (e.g., a glass of water or a vegetable). For alcohol, pictures of beer, champagne, whisky, Ricard^®^, and wine were used. For non-alcohol, pictures of coffee, milk, Coca-Cola^®^, and water were used. For fruits, pictures of apricot, banana, strawberry, orange, green papaya, and apple were used. For vegetables, pictures of carrot, cabbage, endive, and beans were used. Each trials were full random. Each session lasted approximately 2 min for a total 60 trials (250 ms between each trial) and pictures were displayed until participant pressed the screen. Immediately afterward, the participant saw their response time and whether the answer was correct or incorrect (during 500 ms), before new trials (see Figure 1).

### Statistical Analysis

Descriptive and comparative analyses between the two groups of participants were carried out using Student’s *t*-tests. For cognitive scores, analyses were conducted on the average response time given per item for each block when correct answers were given. Responses shorter than 300 ms and longer than 1,500 ms were excluded from the analyses. Each *attentional bias score* was calculated by subtracting the response times for words semantically associated with alcohol and responses times for neutral words. An *attentional performance score* was calculated by subtracting the response times for colored words and the responses times for neutral words. Finally, the Hayling test score was used to assess *inhibitory abilities*. Three ANOVAs on these three variables (Attentional bias score, attentional performance, inhibitory abilities) were performed with a 2 × 2 mixed measures design with a time factor (before or after the 15 days, within-subject) and a participant status factor (alcohol attentional training group vs. control group, between subject).

The significance threshold was set at 0.05, and all analyses were performed using JAMOVI version 1.0 software.

## Results

### Description of the Population

Fifty students participated in the study. Only 47 returned to phase two and were therefore included in the analyses. Twenty participants were randomly included in the cognitive remediation group (mean age: 21.05, SD 2.82; 13 females and 7 males) and 27 in the control group (mean age: 21.44, SD 5.17; 19 females and 8 males). No significant difference was observed between the two groups in age (*p* = 0.759), craving score on the OCDS (14 for the cognitive remediation group vs. 13 for the no remediation group; *p* = 0.323) and AUDIT (9.55 for the cognitive remediation group vs. 7.54 for the no remediation group; *p* = 0.336).

### Evolution of Performance on Cognitive Tests

#### Attentional Bias

**FIGURE 2:**
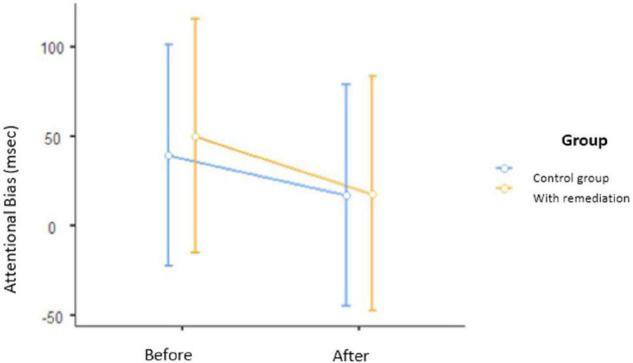
Means and standard deviations of attention bias scores on the Alcohol Stroop Test before the program and after the 15 days of the program.

Our analyses showed no interaction effect of the cognitive remediation program on the reduction of attentional bias [*F*_(1, 44)_ = 0.024, *p* = 0.876, η^2^ = 0.00], no effect of group [*F*_(1, 44)_ = 0.031, *p* = 0.861, η^2^ = 0.00], and no effect of time [*F*_(1, 44)_ = 0.777, *p* = 0.383, η^2^ = 0.008] (see Table 1 and Figure 2).

**TABLE 1:** Means and standard deviations of attention bias scores on the Alcohol Stroop Test before the program and after the 15 days of the program.

				95% CI	
Group	Time of collection	Mean	*SD*	Min.	Max.
Control group	Before	39.7	31.0	−21.9	101.3
	After	17.2	31.0	−44.4	78.8
With remediation	Before	50.3	32.9	−15.1	115.8
	After	18.2	32.9	−47.3	83.6

#### Attentional Performance

Our analyses showed no interaction effect of the cognitive remediation program on enhancement of attentional performance [*F*_(1, 44)_ = 1.63, *p* = 0.208, η^2^ = 0.014], no effect of group [*F*_(1, 44)_ = 0.373, *p* = 0.544, η^2^ = 0.055], and no effect of time [*F*_(1, 44)_ = 0.015, *p* = 0.903, η^2^ = 0.00] (see Table 2).

**TABLE 2:** Means and standard deviations of the scores of the Classic Stroop in the Alcohol Stroop Test before the program and after the 15 days of the program.

				95% CI	
Group	Time of collection	Mean	*SD*	Min.	Max.
Control group	Before	43.5	39.0	–34.07	121
	After	84.6	39.0	7.08	162
With remediation	Before	116.2	41.3	34.04	198
	After	66.3	41.3	–15.86	148

#### Inhibitory Abilities

Our analyses showed no interaction effect of the cognitive remediation program on enhancement of inhibitory abilities [*F*_(1, 44)_ = 0.120, *p* = 0.731, η^2^ = 0.001], no effect of group [*F*_(1, 44)_ = 0.053, *p* = 0.818, η^2^ = 0.011]. We observed an effect of time [*F*_(1, 44)_ = 13.163, *p* < 0.001, η^2^ = 0.101] (see Table 3).

**TABLE 3:** Means and standard deviations of inhibition ability scores measured using the Hayling test before the program and after the 15 days of the program.

				95% CI	
Group	Time of collection	Mean	*SD*	Min.	Max.
Control group	Before	35.1	5.08	25.00	45.2
	After	20.0	5.08	9.89	30.1
With remediation	Before	35.4	5.38	24.66	46.0
	After	17.1	5.38	6.36	27.7

### Bayesian Analyses

Because the probabilistic analyses showed insignificant results, we decided to refine these results with a Bayesian analysis. Bayesian analysis was performed to assess which model provides more support for the null hypothesis. We use the Bayes factor (BF) to compare the probability of the data under a single model to provide evidence in favor of the null hypothesis [BF01, (25)]. We followed a model comparison approach that assesses the added value of each new predictor; in our study, these predictors included the potential effect of attentional bias at Time 2 vs. Time 1. The Bayes exclusion factor reflects the evidence for all models without a particular term compared to all models with that particular term. For example, a Bayes factor exclusion for term A of four means that all models containing term A are four times less likely than models without term A.

All models provided substantial evidence for the null hypothesis. For models with all terms and intra*intermediate interaction, Bayesian analysis found strong evidence against training with attentional bias (BF01 = 38.32), confirmed by the exclusion of the Bayes factor (BFexcl = 16.14). Similar results were obtained for attentional capacity assessed by the Stroop effect (BF01 = 24.19, BFexcl = 16.14) and the Hayling test (BF01 = 11.37, BFexcl = 16.14).

### Analyses According to the Level of Attention Bias at the Program Initiation

An ANOVA carried out only on observations with an initial attentional bias score greater than 0 (28 participants in total: 14 in the cognitive remediation group, and 14 in the control group) clearly showed a reduction in attentional bias [Mean = 92.5, *SD* = 109; Mean_After_ = −51,3, *SD* = 91—*F*_(1, 26)_ = 36.453, *p* < 0.001, η^2^ = 0.356]; however, no group effect [*F*_(1, 26)_ < 0.0001, *p* = 0.977, η^2^ = 0.00] and no interaction [*F*_(1, 26)_ = 0.342, *p* = 0.564, η^2^ = 0.003] were observed. Similar results were observed for the Hayling test, which showed a decrease in response time and thus an increase in attention span [Mean_Before_ = 37.4, *SD* = 23.7; Mean_After_ = 23.9, *SD* = 30.5—*F*_(1, 26)_ = 3.953, *p* = 0.057, η^2^ = 0.07] with no group effect [*F*_(1, 26)_ = 0.005, *p* = 0.0944, η^2^ = 0.000] and no interaction effect [*F*_(1, 26)_ = 0.074, *p* = 0.788, η^2^ = 0.001]. In addition, we did not find any effect of the classical Stroop test [no reduction [*F*_(1, 26)_ = 0.133, *p* = 0.718, η^2^ = 0.002]; no group effect [*F*_(1, 26)_ = 0.00584, *p* = 0.940, η^2^ = 0.000] and no interaction [*F*_(1, 26)_ = 1.524, *p* = 0.228, η^2^ = 0.023]].

For participants with no initial attentional bias (18 participants in total, including six in the cognitive remediation group and 12 in the control group), we observed no effects on performance in the Alcohol Stroop test or the classical Stroop test, but inhibitory function improved in the Hayling test [Mean = 32, *SD* = 18.5; Mean_After_ = 12.5, *SD* = 22.5; *F*_(1, 16)_ = 13.737, *p* = 0.002, η^2^ = 0.214]. for this dimensions, there was no effect of group [*F*_(1, 16)_ = 1.37, *p* = 0.259, η^2^ = 0.042] or any interaction effect [*F*_(1, 26)_ = 0.224, *p* = 0.642, η^2^ = 0.003].

## Discussion

The objective of this pilot study was to evaluate the effectiveness of a smartphone cognitive remediation program for mitigating alcohol attention bias in students. Neither classical, frequentist, nor Bayesian analyses showed a greater decrease in attentional bias for the program group on alcohol-related items. This lack of effect between group was also observed in attentional and inhibitory functions. However, an analysis of participants that showed an attentional bias at the beginning of the study (a total of 29 participants) showed a decrease in attentional bias and an increase in inhibition capacities but not in attentional capacities when we compared score at beginning and at the end of the study. This improvement in attentional bias, however, did not depend on the type of program (remediation or control) used by the participants, indicating a lack of specificity of the application. In summary, this program appears to reduce attentional bias to alcohol regardless of item type. It also seems to contribute to the improvement of inhibition functions. This seems to be in line with the hypotheses of the dual process addiction model. In these models, the addictive problem is due to a dysregulation between an emotional system and a control system (Hofmann et al., 2008). Based on these models, it would seem that a program that improves only the control system (in which inhibition capacities are included) also decreases the attentional bias. Such a program could therefore potentially reduce substance use as well.

Several explanations for explained the lack of specificity of the program using alcohol-related items can be put forward. First, motivation to complete the task was not evaluated. However, many studies now show that motivation is important for change in this type of program (Eberl et al., 2013; Boffo et al., 2019; Flaudias et al., 2019). Therefore, we cannot exclude the possibility that our participants were not sufficiently motivated to perform the task adequately. Including a measure of motivation in future studies is therefore indispensable.

We may also question the duration of the training. Two minutes of training may not be enough to produce results. Indeed, programs with significant results are often longer, such as 30 min in our patient version (Flaudias et al., 2019). Nevertheless, in order to increase student commitment to participate in this program over 15 days, 30 min does not seem to be a realistic choice. It is therefore important in the future to assess the minimum threshold for effect. Additionally, our application could not record the sessions performed by the user for this type of analysis because the release of a new version of Android during the study disabled this initially planned feature. Finally, we may question our tools for measuring attentional bias. Indeed, AST explores two dimensions of attentional bias: early detection of a stimulus and disengagement difficulties. Scores on the Hayling test and the classical Stroop test seemed to indicate (although non-significantly) an increase in attentional and disengagement capacities. The AST may not be sensitive enough. Tasks distinguishing disengagement difficulties (e.g., Attentional Networking Task; Fan et al., 2002) as well as the ability to detect stimuli early (e.g., a dot probe; MacLeod et al., 1986) could make it possible to refine the different dimensions of attentional bias, which could be modulated by a remediation program. In addition, we did not assess alcohol use before and after the program. This criterion could also be relevant to assess the effect of such a program from a behavioral point of view.

These aspects indicate the inherent difficulties in both modifying therapeutic programs for patients with alcohol use disorders and alcohol use prevention in healthy participants.

Finally, we must also be cautious about these conclusions, because we conducted *a priori* power analyses, which seems to be sufficient, and that the Bayesian analyses confirm this absence of effect on the Alcohol Stroop Test, we cannot exclude that the number of participants remain low for this type of study.

Despite the lack of effects, we believe that prevention of alcohol drinking via gamification on smartphones aimed at reducing attentional bias remains an interesting avenue (see Wiers’ comment on this subject; Wiers, 2018). In addition, it is important to continue evaluating these applications, because despite an increase in the number of programs available, very few have been scientifically evaluated and only one has been shown to be effective (Zhang et al., 2018). This is true since we have shown an increase in attentional bias in patients with no initial attentional bias, but who have completed the remediation program (Flaudias et al., 2019). The evaluation of these smartphone applications is essential to ensuring the absence of deleterious effects.

Finally, it is important to note that not all alcohol users may show an attentional bias (see e.g., Flaudias et al., 2019) and that it cannot be considered a unique response to the problem. Similarly, from a processual perspective, attentional bias is only one of many psychological processes that can be linked to excessive alcohol consumption, as other processes may come into play, and it is important that interventions target the processes that are relevant to an individual.

Thus, we recommend that future studies in this area (1) increase participant motivation by making the application more engaging and enjoyable to use; (2) explore the length of training required, such as by increasing the number of daily sessions; (3) conduct the study on a sample composed of heavy and light drinkers to facilitate comparisons of program effectiveness; and (4) use several tools to assess attentional bias to ensure that all dimensions of this cognitive process are measured.

## Data Availability Statement

The raw data supporting the conclusions of this article will be made available by the authors, without undue reservation.

## Ethics Statement

The studies involving human participants were reviewed and approved by the Ethical Committee of Clermont Université. The patients/participants provided their written informed consent to participate in this study.

## Author Contributions

VF, NC-B, and GB: participated to study concept and design, and participated to the study supervision. VF and OZ: to the analysis and interpretation of data and writing the initial draft of the article. VF, OZ, NC-B, ID, P-ML, and GB: reviewed the initial draft and participated in the writing of the final draft. VF, OZ, and NC-B: had full access to all data in the study and take responsibility for the integrity of the data and the accuracy of the data analysis. All authors contributed to the article and approved the submitted version.

## Conflict of Interest

The authors declare that the research was conducted in the absence of any commercial or financial relationships that could be construed as a potential conflict of interest.

## Publisher’s Note

All claims expressed in this article are solely those of the authors and do not necessarily represent those of their affiliated organizations, or those of the publisher, the editors and the reviewers. Any product that may be evaluated in this article, or claim that may be made by its manufacturer, is not guaranteed or endorsed by the publisher.
